# Suitability of traits related to aggression and handleability for integration into pig breeding programmes: Genetic parameters and comparison between Gaussian and binary trait specifications

**DOI:** 10.1371/journal.pone.0204211

**Published:** 2018-12-28

**Authors:** Uta König von Borstel, Björn Tönepöhl, Anne K. Appel, Barbara Voß, Horst Brandt, Saeid Naderi, Matthias Gauly

**Affiliations:** 1 Department of Animal Science, Livestock Production Systems, Georg August University Göttingen, Göttingen, Germany; 2 Institute for Animal Breeding und Genetics, Justus Liebig University Giessen, Giessen, Germany; 3 BHZP GmbH, Dahlenburg-Ellringen, Germany; Humboldt-Universitat zu Berlin, GERMANY

## Abstract

Changes in husbandry systems as well as consumers’ increasing demands for animal welfare lead to increasing importance of traits such as handleability and aggressiveness in pigs. However, before using such novel traits for selection decisions, information on genetic parameters for these traits for the specific population is required. Therefore, weight gain and behaviour-related traits were recorded in 1004 pigs (814 Pietrain x German Landrace crossbred, 190 German Landrace purebred) at different ages. Behaviour indicators and tests were assessed and conducted, respectively under commercial farm conditions and included scoring of skin lesions (twice) and behaviour during backtests (twice), injections (once), handling (twice) and weighing (three times). Since behaviour scores often exhibit suboptimal statistical properties for parametric analyses, variance components were estimated using an animal model assuming a normal (Gaussian, GA; all traits) and additionally a binary distribution of variables (BI; using a logit-link function for all behaviour traits). Heritabilities for behavioural traits ranged from 0.02 ± 0.04 (finishing pig handling test; BI) to 0.36 ± 0.08 (backtest 2; GA) suggesting that some of the traits are potentially useful for genetic selection (e.g. finishing pig weighing test: h^2^ (GA) = 0.20 ± 0.07). Only minor differences were observed for results from binary and Gaussian analyses of the same traits suggesting that either approach might yield valid results. However, four-fold cross-validation using correlations between breeding values of a sub-set of animals for the sample trait finishing pig weighing score indicated slight superiority of the logit model (r = 0.85 ± 0.04 vs. r = 0.77 ± 0.03). Generally, only weak to moderate associations were found between behavioural reactions to the same test at different ages (r_p_ ≤ 0.11 for weighing at different ages; r_p_ = 0.30 but r_g_ (GA) = 0.84 ± 0.11 for the backtests) as well as between reactions to different tests. Therefore, for inclusion of behaviour traits into breeding programmes, and considering high labour input required for some tests such as the backtest, it is recommended to assess behaviour during situations that are relevant and identical to practical conditions, while the use of indicator traits generally does not appear to be a very promising alternative.

## Introduction

Public concern about well-being of farm animals recently increased [1, 2] leading to considerable changes in pig production systems. In response to this increasing public concern, laws including specific requirements for pig housing and welfare (e.g., group housing of pregnant sows or provision of enrichment items) have been passed (2008/120/EG). However, there are also structural changes in livestock farming. Herd sizes increase and new housing systems with higher levels of automation become more and more common [3, 4], leading to reduced human-animal contact and, as a consequence, less habituated animals when handling is necessary. Overall, pig housing systems will increasingly be characterized by intensive farming conditions and group housing, often with increasingly large groups, which inevitably leads to agonistic interactions within the group [5]. In the case of fattening pigs, the demand for highly homogenous body weights at slaughter may lead to frequent disturbance of social relationships within a group due to removal of individual pigs at different points in time as they reach slaughter weight. At the same time human-animal interactions are reduced, providing fewer opportunities for the animals to habituate to the presence of and handling by human beings [6, 7]. As a consequence, the need for inherently calm and easy to handle pigs, with low levels of aggression further increases in order to reduce stress for the animals at handling or mixing procedures, and pig breeding companies now consider changing their breeding goals towards a stronger focus on behaviour traits reflecting these characteristics.

Due to reasonable heritabilities of a number of behaviour and related indicator traits [8–10] the integration of behavioural traits into pig breeding programmes appears to be a reasonable possibility to enhance animal welfare by breeding more docile pigs [11, 12]. Also, associations between different behaviour traits exist, potentially reducing the number of traits that need to be recorded. In some pig populations, aggressive behaviour at mixing is genetically correlated with the response to handling [11]. At the phenotypic level, there are also associations between the number of escape attempts in the backtest and behaviour towards unfamiliar individuals [13, 14] as well as the pigs’ behaviour towards novel objects/situations [2, 15], but these relationships are also dependent on pigs’ age at time of testing, interactions between personality and environment [16], and often were demonstrated only in those studies selecting extreme phenotypes [14, 17] rather than all pigs [17]. Over a short period of time pigs respond consistently to a specific situation or social challenge [18, 19], including general aggressiveness during feeding [17], but not when the challenge occurred in a different context [20]. Thus, a short-term consistency of certain behaviour patterns appears to be existent, and different behaviour traits may partially share a common genetic background. However, the results of earlier studies [8–10] also suggest associations between behaviour and performance traits, which have to be estimated and carefully considered before implementation into breeding programmes to minimize unwanted side-effects of selection on behaviour traits. Therefore, the aim of the present study was to estimate heritabilities as well as phenotypic and genetic correlations between fattening pigs’ behaviour over the course of their life during standardized handling tests, skin lesion scores and weight gains to identify traits of interest for inclusion in breeding programmes. In addition we assessed the influence of using binary versus Gaussian approaches to data analysis, as well as the influence of different housing conditions commonly found in practice.

## Animals, materials and methods

### Ethics statement

This type of non-invasive, behavioural research is approved under the German animal protection act and does not require a study-specific permission. All procedures corresponded to or were similar to routine handling and management practices commonly occurring in pig farming.

### Animals and housing

The investigation was part of a larger study considering all stages of pig production. The present study was concerned with fattening pigs throughout their life and carried out using 1004 pigs. Among them were 814 Pietrain x German Landrace crossbreds (418 castrates and 396 females) and 190 German Landrace purebreds (97 castrates and 93 females). These breeds were chosen as they represent each a dam and boar line of two of the three most commonly used breeds for commercial crossbreeding in Germany. Due to piglet losses and technical problems, not all pigs had records for all traits, resulting in reduced sample sizes for some traits (backtest 1: n = 1004, backtest 2: n = 769, injection score: n = 769, piglet scale score: n = 874, rearing pig load score: n = 987, rearing pig scale score: n = 987, finishing pig load score: n = 976, finishing pig scale score: n = 976, skin lesion score 1: 1004, skin lesion score 2: 987, daily gain, suckling period: n = 996, daily gain rearing period: n = 777, daily gain finishing period: n = 603, daily gain lifetime: n = 603). The pigs descended from 135 sows and 31 boars. Pedigree data included two generations back, resulting in a total of 1.688 animals in the pedigree.

The animals were housed under commercial farm conditions at the research farm of the University of Göttingen. A three-weekly batch farrowing rhythm (i.e. sows are synchronized so that farrowing takes place every three weeks) with 28 days of lactation was followed, during which piglets were kept in conventional farrowing pens with fully slatted floors and creep feeders. First, the piglets were penned with their littermates in the farrowing pen with fully perforated plastic floors. The total space allowance in the farrowing pen was 5 m^2^ including the farrowing crate of the sow and the creep area (0.65 m^2^). According to German typical pig farming procedures, iron injections, tail docking and castration of male piglets were conducted during the piglets’ first 4 d after birth. Furthermore, each piglet was tattooed on the right ear for individual identification. At weaning, the pigs were additionally marked by an ear tag, before the animals were moved into the rearing pens which were equipped with fully perforated floors. In the rearing pens, group sizes were either 10 (3.57 m^2^), 30 (11.1 m^2^) or 41 (15.5 m^2^) animals. Thus, piglets in the larger pens were mixed with random, convenience samples (i.e. whichever piglets of similar age were available at the same time) of unfamiliar piglets from additional litters, to fill up the pen. Pens were enriched either with a plastic star on a chain or a piece of wood on a chain. Per every 10 animals in the pens there was one enrichment item. The feeding systems included both dry feeders and wet-dry feeders and drinking nipples. Rearing ensued up to a body weight of 40 kg. Pigs were then moved into the finishing barn, where they were randomly sorted into groups of 12 (9.3 m^2^), 24 (18.6 m^2^), and 36 (27.9 m^2^) animals, or in a large group of 120 (75.9 m^2^) pigs with automatic sorting and feeding systems. Due to the nature of the automatic sorting system, pigs were removed from the group at irregular intervals, so that space requirements were met also towards the later phases of the observations. In both systems dry feeders and drinking nipples as well as fully slatted concrete floors were installed. Plastic stars on a chain were available as enrichment items. The finishing pigs were slaughtered at a live weight of approximately 115 kg. Water and feed was available ad libitum for pigs during the fattening period with the exception of the small groups in the finishing barn. In the latter case, the pigs were fed twice a day. Production information was obtained from the farm logbook. This information included daily gains for the preweaning (PDG), rearing (RDG), and finishing period (FDG) as well as lifetime average daily gain (ADG). There were no outbreaks of diseases or tail-biting observed, in the pigs included in the present research.

### Behavioural observations

All behavioural observations, and scoring of the skin lesions, and all associated handling with the exception of administrating the iron injections, were done by one person. Tests and evaluation systems were designed to cause a minimum of labour costs and disturbance of the daily workflow to meet requirements of commercial farming and breeding, and were pre-selected out of a wider range of tests, based on sufficient inter-observer agreement as well as variance between individuals ([21], unpublished data). Therefore, behavioural observations were integrated into everyday procedures (e.g. weighing or injection of iron supplements).

The backtest was performed first between 1 to 4 d of age (BT1) and again between 15 to 19 d of age (BT2), i.e. for each piglet 14 d after the first backtest. For this procedure, piglets were separated from their dam. Then each piglet was individually turned on its back and restrained in this supine position in the experimenter’s right hand (adapted from [13]). For time-efficiency, the time frame for the backtest was reduced to 30 seconds compared to the more commonly used 60 seconds. The total number of attempts to escape was recorded. Each series of struggling without a break was counted as one escape attempt. Piglets’ behaviour during the injection test (INJ) and during weighing as well as the skin lesions were recorded using behaviour scores as described in Table 1. The newly developed INJ score was assigned during routine iron injection at day 1–4 by assessing the intensity of piglets’ squeals and struggling while the piglet was held for a duration of approximately 1 second in one hand and while receiving an intramuscular iron injection in the neck. At weaning the behaviour of the animals on a weighing scale for piglets (Sartorius, Göttingen, Germany) was observed for 30 seconds and evaluated with a score from 1 to 3 regarding the amount and intensity of locomotor behaviour (piglet scale score, PSS; Table 1). A second weighing was done when moving the pigs from rearing pens into the finishing barn using a commercial pig scale (Texas Trading, Windach, Germany). During this handling procedure, the behaviour of each pig was scored twice: (a) while the handler placed the pig onto the weighing scale (rearing pig load score, RLS) and (b) during its first 30 s on the scale (rearing pig scale score, RSS). Both scores rated the pig's agitation from low (1) to high (3) (Table 1). The extent of skin lesion and wounds was evaluated using a score from 1 to 4 (lesion score 1, LS1) (Table 1) after assessment of RSS and immediately before moving the pigs further to the finishing barn. The recording of the skin lesion scores was rerun 24 h post mixing (lesion score, 2, LS2) and the difference between LS1 and LS2 calculated (lesion score difference, LSD) in order to obtain an estimate of the amount of lesions originating from fights during mixing. Before transport to slaughter, the finishing pigs were weighed for the last time. Test procedures were comparable to the second weighing, including a behaviour score for loading onto the scale (fattening pig load score, FLS) and while weighing (fattening pig scale score, FSS). An overview of the time-line of the experimental procedures is shown in Table 2.

**Table 1:** Overview of handling procedures (adapted from [22, 23]) and indicators of aggressiveness (adapted from [2, 24]), definition of respective scores for assessment and scheme for re-coding as binary trait.

Behaviour trait	Score	Binary	Definition
Handling (rearing and finishing pig scale loading scores)	1	0	The pig enters the scale without hesitation
	2	1	The pig hesitates briefly (total of < 5 sec) before entering
	3	1	The pig refuses to enter; tries to escape before entering
Weighing (piglet, rearing pig, and finishing pig scale score)	1	0	Calm; no/very little movements
	2	1	Excited; slow movements (e.g,. walking)
	3	1	Very excited; fast movements (e.g. jumping, running)
Treatment (injection score)	1	0	Calm; no vocalisation or struggling
	2	0	Brief vocalisation and struggling (i.e. 1–2 brief squeals and/or struggling bouts)
	3	1	Frequent vocalisation and struggling for about 50% of test duration
	4	1	Permanent, high-pitched vocalisation and struggling
Aggressiveness (skin lesion score 1 + 2)	1	0	No lesions
	2	0	Several lesions (i.e., less than 5 scratches of less than 10 cm length each)
	3	1	Many distinct lesions (i.e., 5 to 10 scratches of < 10 cm or at least one scratch of > 10 cm length)
	4	1	Wounds, lesions all over the body (> 10 scratches)

**Table 2:** Time-line of the experimental procedures.

Area	Farrowing						Rearing		Finishing	
Week	0	1	2	3		4	5–10	11–15	16–22	23–27
Procedure	Birth, Preweaning				Weaning, Rearing			Fattening		
Behaviour		BT1 INJ		BT2		PSS		RLS RSS LS1	LS2	FLS FSS

BT1: backtest 1 between 1 to 4 d of age; BT2: backtest 2 14 days after BT1, i.e. between 15 to 19 d of age; INJ: injection score between 1 to 4 d of age; PSS: piglet scale score; RLS: rearing pig load score; RSS: rearing pig scale score; LS1: skin lesion score before mixing; LS2: skin lesion score 24 h post mixing; FLS: finishing pig load score; FSS: finishing pig scale score.

### Statistical analyses

The data from behaviour tests and the production information were analysed with mixed models using the models described below and the statistical software package SAS (SAS 9.3; software SAS Institute Inc., Cary, NC; 2002–2010). Age and weight at testing were likewise tested for their effects on behaviour variables, but not considered in the further genetic analysis because of low and in most cases insignificant influences at the phenotypic level. Due to the commercial conditions, animals were housed in different husbandry systems both during rearing and finishing. Therefore, and based on the results of an earlier study conducted on the same farm [25], the type of housing (defined as larger or smaller pens with correspondingly smaller or larger group sizes and additional feeders, drinkers, and enrichment items in larger pens; see chapter “animals and housing”) was included in the models when analyzing the traits recorded during the rearing or finishing period. The extent of pigs’ skin lesions post mixing is mainly influenced by the number of unacquainted pigs resulting in fights for a new social order [1, 5]. Therefore, four classes were generated per pig according to the number of unacquainted pigs (no unfamiliar pig; 1–10 unfamiliar pigs; 11–30 unfamiliar pigs; more than 30 unfamiliar pigs). The Model for ADG included the type of housing during rearing as well as during finishing. All production traits were treated as linear variables. All behaviour traits were considered in separate runs as either Gaussian or binary variables. In the latter case, scores were re-coded as either 0 or 1 as outlined in Table 1. For the results from the backtests, two or fewer attempts to struggle were coded as 0 while 3 and more attempts to struggle were coded as 1. Genetic parameters were estimated using uni- and bivariate models and the software DMU 6 version 5.2 [26], assuming either Gaussian distributions or using a logit-link function for analyses assuming binary distributions.

Based on the results of the phenotypic analyses, the genetic models for behaviour as well as performance traits included the following effects:

BT1, BT2, INJ, PSS, PDG (preweaning period):

$$y_{ijkl}=\mu +{sex}_{i}+{breed}_{j}+{litter}_{k}+{pig}_{l}+e_{ijkl}$$

RLS, RSS, LS1, RDG (rearing period):

$$y_{ijklm}=\mu +{sex}_{i}+{breed}_{j}+{rearing}_{k}+{litter}_{l}+{pig}_{m}+e_{ijklm}$$

LS2, LSD (finishing period):

$$y_{ijklmn}=\mu +{sex}_{i}+{breed}_{j}+{rearing}_{k}+{familiar}_{l}+{litter}_{m}+{pig}_{n}+e_{ijklmn}$$

FLS, FSS, FDG (finishing period):

$$y_{ijklm}=\mu +{sex}_{i}+{breed}_{j}+{finishing}_{k}+{litter}_{l}+{pig}_{m}+e_{ijklm}$$

ADG (lifetime):

$$y_{ijklmn}=\mu +{sex}_{i}+{breed}_{j}+{rearing}_{k}+{finishing}_{l}+{litter}_{m}+{pig}_{n}+e_{ijklmn}$$

where y indicates the observation for pig’s reaction in the behaviour tests, skin lesions or performance traits; sex is the fixed effect of the gender with two classes (barrow/gilt); breed is the fixed effect of the genetic line with two classes (German Landrace / Pietrain x German Landrace); rearing is the fixed effect of the housing type in the rearing quarters (three classes of different pen sizes); finishing is the fixed effect of the housing type in the finishing barn (two classes of different compartment sizes); familiar is the fixed effect of the number of unfamiliar pigs post mixing (four classes); litter is the random permanent environmental effect of the litter; pig is the random effect of the animal; and e is the residual effect. In order to estimate genetic and phenotypic correlations in bivariate analysis, the following model was applied:

$$\left[\begin{array}{c} y_{1}\\ y_{2} \end{array}\right]=\left[\begin{array}{cc} X_{1} & 0\\ 0 & X_{2} \end{array}\right]\left[\begin{array}{c} b_{1}\\ b_{2} \end{array}\right]+\left[\begin{array}{cc} P_{1} & 0\\ 0 & P_{2} \end{array}\right]\left[\begin{array}{c} l_{1}\\ l_{2} \end{array}\right]+\left[\begin{array}{cc} Z_{1} & 0\\ 0 & Z_{2} \end{array}\right]\left[\begin{array}{c} a_{1}\\ a_{2} \end{array}\right]+\left[\begin{array}{c} e_{1}\\ e_{2} \end{array}\right]$$

where *y*_*i*_ is vector of records in *i*th trait (*i* = 1 and 2); *b*_*i*_, *l*_*i*_, *a*_*i*_, and *e*_*i*_ are fixed, permanent environmental, direct additive genetic and residual effects for *i*th trait, respectively. *X*_*i*_, *P*_*i*_ and *Z*_*i*_ are the design matrices associating the corresponding effects.

The (co)variance structure for random effects was:

$$var\left[\begin{array}{c} l\\ a\\ e \end{array}\right]=\left[\begin{array}{ccc} {I_{l}\sigma }_{pe}^{2} & 0 & 0\\ 0 & {A\sigma }_{a}^{2} & 0\\ 0 & 0 & {I_{n}\sigma }_{e}^{2} \end{array}\right]$$

Where, *l* is the permanent environmental effect related to litter size, *a* is the direct additive genetic effect and *e* is the residual effect; $\sigma_{pe}^{2}$ is the permanent environmental variance; $\sigma_{a}^{2}$ is the direct additive genetic variance and $\sigma_{e}^{2}$ is the residual variance; **A** is the additive numerator relationship matrix; ***I***_*l*_, ***I***_n_ are the identity matrices with order equal to the number of litter size groups (*l*) and records (*n*), respectively. For comparisons, heritabilities from the logit model were additionally transformed according to [27].

Additionally, for the sample trait FSS, a cross-validation approach was used to compare predictive abilities of the binary and the Gaussian model. For this purpose, the data set was split into four sub-groups of 251 animals each, and using both models breeding values were estimated for all animals using all records and subsequently using phenotypic records from only 3 of the 4 sub-sets, while predicting breeding values for the remaining subset. This was repeated for each sub-set, and the entire procedure was repeated five times. For each replicate, Pearson correlation coefficients were calculated between breeding values calculated for the respective subset of animals with and without considering their phenotypic records in breeding value estimation, and they were subsequently summarized per model as mean correlation ± SD.

## Results

Phenotypic means, standard deviations, minima, and maxima of the behaviour and performance traits are presented in Table 3.

**Table 3:** Phenotypic mean, standard deviation (SD), minimum, maximum, of behaviour and performance traits.

Trait	Mean ± SD	Minimum	Maximum
Backtest 1	2.4 ± 1.2	0	8
Backtest 2	2.5 ± 1.2	0	9
Injection score	2.8 ± 0.8	1	4
Piglet scale score	2.0 ± 0.8	1	3
Rearing pig load score	1.8 ± 0.8	1	3
Rearing pig scale score	2.0 ± 0.8	1	3
Finishing pig load score	1.5 ± 0.7	1	3
Finishing pig scale score	1.5 ± 0.7	1	3
Skin lesion score 1	2.3 ± 0.8	1	4
Skin lesion score 2	2.9 ± 0.9	1	4
Daily gain suckling period	224 ± 53	29	404
Daily gain rearing period	413 ± 67	191	651
Daily gain finishing period	760 ± 104	339	1096
Daily gain lifetime	600.± 53	435	744

Backtest: number of attempts to struggle; Weighing behaviour (load / scale): score 1–3; Behaviour during injection: score 1–4; Skin lesions: score 1–4; Daily gains: grams.

**Fig 1:**
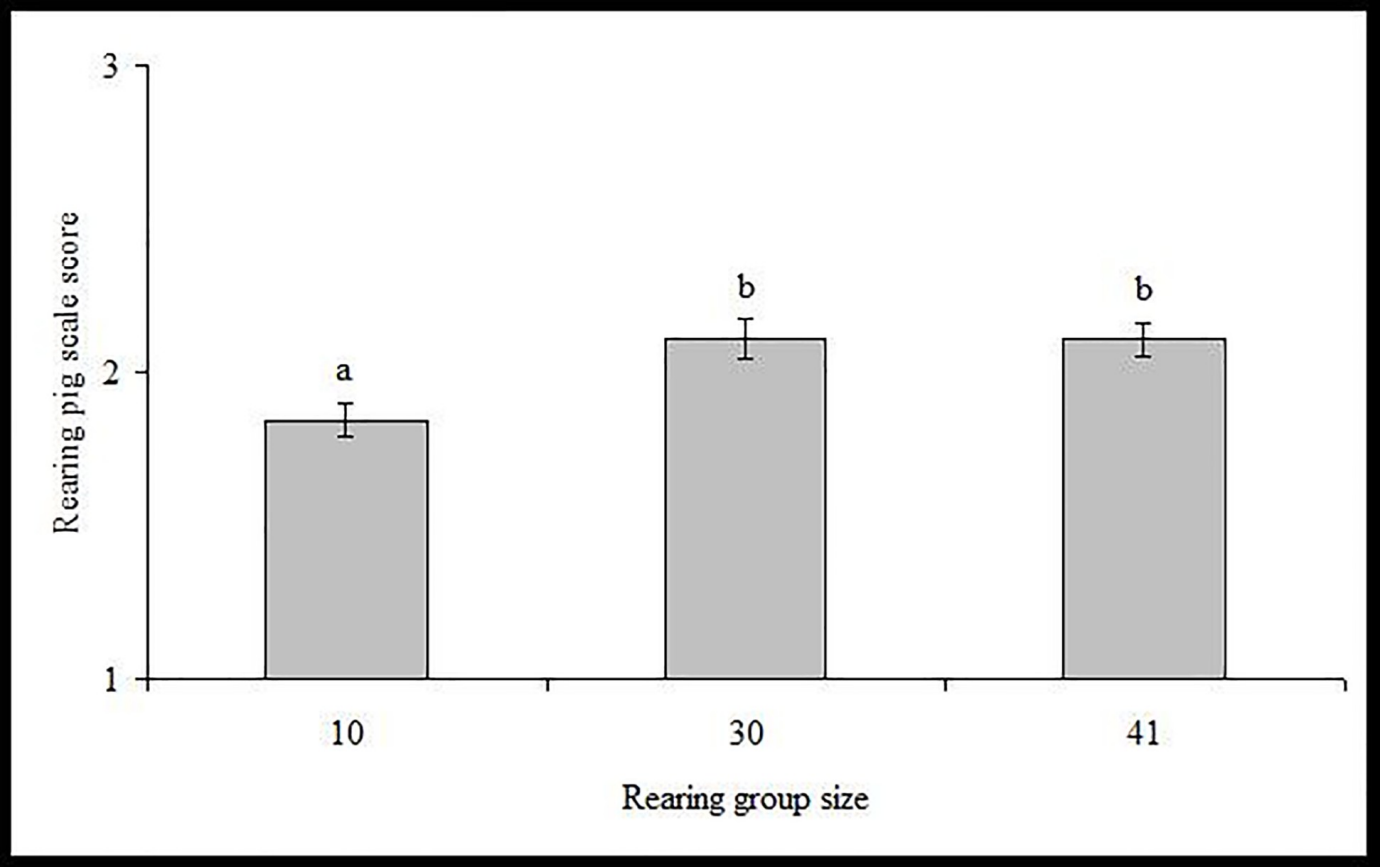
Effect of group size (10, 30, or 41 individuals) including different housing types (more total space, feeders, drinkers and toys as group size increases) during rearing on rearing pigs’ scale scores. (Larger scores indicate increased agitation; different letters indicate significant differences at p < 0.001).

The type of housing system affected the behaviour of pigs. Fig 1 shows the effect of rearing housing system on RSS. Rearing pigs housed in groups of ten animals were more passive while being weighed and were thus scored with significantly lower scores compared to animals of larger groups. However, this effect was not seen during handling while loading the rearing pigs onto the scale (RLS: P > 0.05). Pigs housed in the smallest groups had initially (LS1) slightly higher lesion scores (2.3 ± 0.05) compared to those housed in the medium (2.1 ± 0.04) and large groups (2.1 ± 0.05; both P < 0.05). However, after mixing, pigs of the large groups had higher lesion scores compared to pigs of the small group (LS2: 2.8 ± 0.06 vs. 2.6 ± 0.08), with pigs of the medium sized groups ranking in the middle (2.7 ± 0.06). As a consequence, LSD was higher for pigs of the two larger groups (both 0.7 ± 0.06) and lower for pigs of the small group (0.3 ± 0.07; both P < 0.0001). Similarly, pigs housed in the large group for finishing, had higher lesion scores after mixing compared to pigs housed in the smaller groups for finishing (LS2: 2.7 ± 0.05 small groups vs 3.0 ± 0.08 in the large group; P < 0.0001). Pigs housed in the large group for finishing had lower load and scale scores (FLS: 1.3 ± 0.05 vs. 1.5 ± 0.03; P = 0.0003; FSS: 1.4 ± 0.05 vs. 1.6 ± 0.03; P = 0.0034) than those housed in smaller groups. Considerable differences were also observed for the daily gain between the housing systems: Pigs of the small groups (395 ± 5.4 g) had lowest RDG, compared to pigs of the medium (408 ± 3.9 g; P = 0.06), and in particular as compared to the large groups (445 ± 4.5g; P < 0.0001), but effects of housing were not seen during the finishing period (FDG: P > 0.05).

### Heritabilities and model comparison

Variance components from linear and logit threshold models are presented in Table 4. For a number of traits (i.e., INJ, PSS, RLS, FLS, PDG, RDG) the additive genetic variance was very low, and based on the high standard errors, heritabilities for these traits were not significantly different from zero. Furthermore, as a result of the low additive genetic variance, genetic correlations of these traits towards other traits were commonly near 1 or –1. Therefore, with the exception of the trait INJ, for which no analogous behaviour traits were recorded at other stages of pigs’ life, the results of these traits were not included in Tables 5 and 6.

**Table 4:** Estimates of additive genetic variance (σ2A), common environmental litter variance (σ2LE), residual variance (σ2e) and heritability (h^2^) for behaviour and performance traits using a linear animal model and using a threshold (logit) animal model including estimates of h^2^ transformed (h2tr) scale according to [27].

	linear model				threshold model (σ^2^_e_ = 3.29)			
Traits	σ^2^_A_	σ^2^_LE_	σ^2^_e_	h^2^	σ^2^_A_	σ^2^_LE_	h^2^	h^2^_tr_
**Behaviour traits**								
Backtest 1 (BT1)	0.32	0.12	1.04	0.22 ± 0.10	0.47	0.11	0.12 ± 0.06	0.21
Backtest 2 (BT2)	0.52	0.08	0.87	0.36 ± 0.08	0.40	0.27	0.10 ± 0.05	0.53
Injection score (INJ)	0.03	0.13	0.34	0.07 ± 0.07	0.00	0.47	0.00 ± 0.06	0.08
Piglet scale score (PSS)	0.04	0.13	0.46	0.07 ± 0.07	0.17	0.21	0.05 ± 0.06	0.05
Rearing pig load score (RLS)	0.05	0.04	0.55	0.04 ± 0.06	0.17	0.26	0.05 ± 0.04	0.18
Rearing pig scale score (RSS)	0.07	0.02	0.48	0.12 ± 0.05	0.30	0.27	0.08 ± 0.05	0.10
Skin lesion score 1 (LS1)	0.08	0.05	0.43	0.14 ± 0.06	0.33	0.39	0.08 ± 0.05	0.17
Skin lesion score 2 (LS2)	0.07	0.09	0.45	0.12 ± 0.07	0.20	0.55	0.05 ± 0.05	0.32
Lesion score difference (LSD)	0.05	0.02	0.34	0.13 ± 0.06	0.26	0.21	0.07 ± 0.06	0.19
Finishing pig load score (FLS)	0.02	0.00	0.44	0.02 ± 0.03	0.06	0.00	0.02 ± 0.04	0.03
Finishing pig scale score (FSS)	0.09	0.00	0.33	0.20 ± 0.07	0.44	0.05	0.12 ± 0.05	0.65
**Performance traits**								
Preweaning period daily gain (PDG)	112.1	1120.8	1686.8	0.04 ± 0.07				
Rearing period daily gain (RDG)	79.5	1078.8	1717.6	0.03 ± 0.06				
Finishing period daily gain (FDG)	5707.2	1234.3	2859.2	0.58 ± 0.15				
Average daily gain lifetime (ADG)	1228.5	313.5	1010.8	0.48 ± 0.14				

**Table 5:** Estimates of genetic correlations (above diagonal), heritabilities (on diagonal), and phenotypic correlations (below diagonal) assuming Gaussian distributions of selected behaviour traits.

		Handling				Aggressiveness		
	BT1	BT2	INJ	PSS	RSS	FSS	LS1	LS2
BT1	**0.22 ± 0.10**	0.84 ± 0.11	0.10 ± 0.60	–0.24 ± 0.53	0.29 ± 0.30	–0.44 ± 0.30	–0.03 ± 0.33	–0.03 ± 0.11
BT2	0.30	**0.36 ± 0.08**	-0.10 ± 0.56	–0.48 ± 0.65	0.57 ± 0.21	0.05 ± 0.20	–0.01 ± 0.22	–0.46 ± 0.30
INJ	0.22	0.06	**0.05 ± 0.07**	0.64 ± 0.64	-0.90 ± 0.33	-0.26 ± 0.54	0.19 ± 0.67	0.96 ± 0.33
PSS	0.03	0.04	0.05	**0.07 ± 0.07**	0.63 ± 0.67	0.26 ± 0.41	1.00 ± 0.01	0.41 ± 0.49
RSS	0.04	0.13	-0.01	0.08	**0.12 ± 0.05**	0.12 ± 0.27	0.41 ± 0.31	–0.44 ± 0.34
FSS	–0.01	0.03	-0.04	0.06	0.11	**0.20 ± 0.07**	0.25 ± 0.30	–0.02 ± 0.33
LS1	–0.03	–0.03	-0.06	0.01	0.04	–0.03	**0.14 ± 0.06**	0.56 ± 0.25
LS2	–0.03	–0.05	-0.01	0.02	–0.03	–0.02	0.64	**0.12 ± 0.07**

BT1: backtest between 1 to 4 d of age; BT2: backtest between 15 to 19 d of age; INJ: injection score; PSS: piglet scale score; RSS: rearing pig scale score; FSS: finishing pig scale score; LS1: skin lesion score before mixing; LS2: skin lesion score 24 h post mixing.

**Table 6:** Genetic correlations between selected behaviour and performance traits.

	Handling					Aggressiveness		
	BT1	BT2	INJ	PSS	RSS	FSS	LS1	LS2
PDG	–0.61 ± 0.92	–0.21 ± 0.60	0.95 ± 0.14	1.00 ± 0.01	0.71 ± 0.77	0.41 ± 0.76	–0.37 ± 0.91	–0.61 ± 1.63
RDG	0.15 ± 0.65	–0.70 ± 0.28	0.78 ± 0.40	1.00 ± 0.01	1.00 ± 0.01	0.33 ± 0.53	1.00 ± 0.01	0.33 ± 0.55
FDG	–0.07 ± 0.06	–0.05 ± 0.20	-0.27 ± 0.61	0.50 ± 0.42	0.20 ± 0.25	0.14 ± 0.21	0.43 ± 0.25	0.31 ± 0.29
ADG	0.06 ± 0.24	–0.27 ± 0.21	-0.41 ± 0.67	0.67 ± 0.49	0.17 ± 0.26	0.24 ± 0.22	0.33 ± 0.27	0.33 ± 0.29

BT1: backtest between 1 to 4 d of age; BT2: backtest between 15 to 19 d of age; INJ: injection score; PSS: piglet scale score; RSS: rearing pig scale score; FSS: finishing pig scale score; LS1: skin lesion score before mixing; LS2: skin lesion score 24 h post mixing.

Heritabilities for behavioural traits ranged from 0.00 ± 0.03 to 0.36 ± 0.08 on the linear scale and from 0.00 ± 0.06 to 0.12 ± 0.05 on the liability scale, and were for all of the traits of similar magnitude or higher on the transformed scale compared to the original linear scale. Heritability estimates for weight gain ranged between h^**2**^ = 0.03 ± 0.06 (RDG) and h^**2**^ = 0.57 ± 0.13 (FDG). The cross-validation approach yielded a mean (± SD) correlation of breeding values of r = 0.85 ± 0.04 for the logit model and r = 0.77 ± 0.03 for the Gaussian model.

### Correlations between behaviour traits

Phenotypic correlations between behaviour traits are presented in Table 5. Between LS1 and LS2 (r_p_ = 0.64) a part-whole relationship exists because lesions that existed during evaluation of LS1 can be expected to be present to almost the same degree during evaluation of LS2. For the other behaviour traits assessed repeatedly, low levels of consistency across time were observed: of these, the highest phenotypic correlation was found between BT1 and BT2 (r_p_ = 0.30), and the scale scores assessed at different ages (PSS, RSS, FSS) were likewise slightly positively correlated (Table 5). Overall, the phenotypic correlations between the different behaviour traits were low. The strongest associations were present between INJ and BT1 (r_p_ = 0.22), followed by BT2 and RSS (r_p_ = 0.13) and RLS and RSS (r_p_ = –0.14; Table 5).

Table 5 also shows the genetic correlations between different behaviour traits. Due to the high standard errors, results are frequently not different from zero (Table 5). However, genetic correlations between BT1 and BT2 were r_g_ = 0.84 ± 0.11. Other exceptions include BT2 and RSS (r_g_ = 0.57 ± 0.21), LS1 and LS2 (r_g_ = 0.56 ± 0.25), and RLS and RSS (r_g_ = -0.45 ± 0.31). Genetic correlations of PSS with other traits have to be seen with caution due to extremely low additive genetic variance of PSS (Table 4).

### Correlations between behaviour and performance traits

Phenotypic correlations between behaviour traits and weight gain were generally lower than r_p_ = 0.10. Larger phenotypic correlations were only observed between LS1 and PDG (r_p_ = 0.10) as well as RDG (r_p_ = 0.15).

For the most part, genetic correlations showed high standard errors (Table 6), allowing limited conclusions to be drawn from these results. Also, due to low additive genetic variance of PDG, RDG, INJ and the scale scores (Table 4) the values for the genetic correlations with these traits have to be seen with caution (e.g., INJ and PDG: r_g_ = 0.95 ± 0.14). However, there was a tendency that pigs with more severe skin lesions in the end of the rearing period gained more weight (e.g., FDG and LS1: r_g_ = 0.43 ± 0.25.)

## Discussion

### Effects of housing on behaviour, skin lesions and performance

In the present study, a number of different housing systems commonly found in practice were included, and while it goes beyond the scope of the present study, to investigate the details of which factors (i.e., group size, space availability, feeding system etc.) affected pig’s behaviour to which extent, results highlight the importance of taking such factors into account when analysing behaviour traits. In the case of scale and load scores, differences in results of behaviour tests for pigs from different housing systems were generally small, leaving it open to debate whether some of these differences are truly biologically significant. Nevertheless, it is striking that these effects of housing system are frequently observed also in other studies, even if only seemingly marginal differences between systems are investigated [25, 28]. In the present study, in particularly LSD, which is thought to mainly reflect the effect of fights during mixing, differed between pigs housed in different systems, and were of biologically relevant magnitude, with higher LSD with pigs housed in the larger group during finishing. Thus, pigs housed in the large group appeared to be more active in agonistic social interactions during mixing, but more passive during the later handling tests (FLS and FSS). While the former result can be attributed to the larger number of pigs encountered in the large group, it is less clear, why finishing pigs, but not the rearing pigs, of the large group were calmer during handling. Possibly, the differences in feeding regimens (restrictive feeding in small finisher groups vs. ad libitum feeding in large finisher groups) caused the pigs of the large groups to behave more passively compared to the pigs of the small groups.

### Suitability of behaviour tests

Individual differences in behavioural characteristics were shown by previous studies using a number of standardised tests. However, generally there was only a short-term [2, 20], or no (as reported in [29]) consistency in individual behaviour. The association between behaviour in backtests at different ages in the present study (r_p_ = 0.30) was similar to the findings of previous studies, in spite of the reduced time-frame as well as differences in fixating the pigs (hand versus solid surface) for the test in the present study. These comparable results for repeatabilities in studies using different time-frames and different fixation methods for the test suggest that a reduction of the originally arbitrarily chosen test-time and change in fixation method does not reduce the validity of the test. Van Erp-van de Kooij et al. [15] found between different backtests at 3 d and 17 d of age a correlation of r_p_ = 0.31 (p < 0.001), and Zebunke et al. [30] a repeatability of r = 0.27 for tests conducted at the ages of 5, 12, 19, and 26 days. However, when the first backtest is conducted slightly later in piglets’ lives, repeatabilities tend to be higher (day 12 and day 19: r_p_ = 0.31–0.43, [31]; day 10 and day 17: r_p_ = 0.48, p < 0.001, [15]; or between 6 and 10 d of age and again between 13 and 17 d of age: r_p_ = 0.49; [32]), perhaps indicating that piglets’ behaviour recorded very shortly after birth is influenced to a larger extent by environmental conditions (e.g. level of vigour, time since last suckling bout), compared to later ages. This assumption is confirmed by the fact that some other behaviour traits recorded relatively early in piglets’ life such as the injection score or the piglet scale score also show low additive genetic variances and comparably large litter environment variances (see Table 4).

Taken together, the results indicate only a moderate, short-term consistency regarding the total number of attempts to escape which is confirmed by our findings (BT1 and BT2: r_p_ = 0.30; r_g_ = 0.84 ± 0.11) and which is not surprising, considering that behaviour is, *per definitionem*, flexible. For example, it seems to be adaptive for pigs to try an alternative behavioural strategy (either more or less struggling) during a second encounter of a test situation, if a given strategy (e.g., vigorous struggling) was unsuccessful for escaping from the first test situation, leading to comparably low repeatabilities for this trait. Using different measurements such as e.g., latency to or total duration of struggling rather than the number of attempts to struggle as was the case in present study yields comparable results [33] and thus not appears to be specific to just one parameter. Overall, even when taken the natural plasticity of behaviour into account, these correlations are surprisingly low, considering that there is only a short period between the repeated tests, which might indicate that major developmental changes take place during this period. Indeed, other studies did not find associations between backtest parameters [17, 29, 34].

In spite of low repeatabilities, piglets are commonly divided into passive or active coping style using the observations from backtest [13, 15, 17], and based on the assumption that the backtest may serve as general predictor of coping style or temperament, relationship to other challenging situations such as human approach, novel object [9, 15] or resident-intruder tests [9] have been proposed, but not unanimously confirmed [29]. From a statistical perspective, dividing behavioural variation into just two categories (and possibly using only the extreme phenotypes, i.e. disregarding a part of the population that does not fit into the proposed model) rather than using the continuous variation seen at the phenotypic level, results in loss of information and in potentially poorer agreement with the assumptions made regarding the genetic architecture of that trait. In the present study the strongest correlation between different behaviour tests was found between BT2 and RSS (r_p_ = 0.13), indicating that piglets, which struggled more frequently during the backtest, also tended to be more active on the scale. However, this association was only found for the tests at the specific points in time and phenotypic relationships were negligible among the other backtest, handling and aggressiveness tests (Table 5). Given the large number of behaviour tests investigated in the present study, the significant result might as well be due to chance.

Furthermore, in our study test time for backtest was 30 s per piglet that led to a duration of 6 minutes for a litter of 12 piglets. Given that the back test procedure is hardly compatible with typical handling procedures such as iron injections which take only a fraction of this time, under commercial conditions this extra time will hardly be available. Thus, the labour and time input to perform even the modified backtest (30 rather than 60 sec) is very high relative to the limited benefits that can be expected from the incomplete correlations to potentially important traits such as RSS. Possibly, a further reduction of test time could lessen the problem, but correlations to the original backtest need to be assessed, and overall, based on the limited repeatabilities as well as the low correlations to other behaviour traits, the usefulness of the backtest as an indicator for pigs’ general personality or pigs’ behaviour in other situations appears to be rather limited. There were also no correlations between scale scores at different ages. In earlier studies the authors suggested that different tests measured different dimensions of personality [15]. The individual reaction of animals might be affected by situation (test), but also habituation or altered motivation are important factors [35]. However, habituation *per se* should not affect correlations or repeatability of test results as correlations-based repeatabilities are measures of variance rather than absolute agreement. Therefore, if the degree of habituation is consistent across pigs, repeatabilities will nevertheless be high, if pigs showing strongest reaction in the earlier tests also show strongest reactions in later tests relative to the other pigs, as is the case, for example with horses’ reaction to novel objects [36].

Earlier results for consistency in behavioural traits varied between studies [2, 15, 20]. However, animals’ response towards handling seems to be comparably consistent, with high levels of consistency across tests found e.g., for cattle in restraint tests [22] and horses in novel object tests conducted under a rider (r = 0.69–0.75 across time; [36]). With pigs, some studies likewise detected high correlations across different handling situations (e.g., ease of transit vs. response to sudden human approach: r_p_ = 0.44, p < 0.001; [37]). However, our present results could not support the findings of high levels of consistency within or across different handling situations, as evidenced by very low correlations among the scale scores at different ages (PSS, RSS, FSS) (Table 5). Similarly, correlations across tests (e.g., RLS and RSS: r_p_ = –0.14 and r_g_ = –0.45 ± 0.31, showing that pigs which refused to enter the scale were less agitated on the scale), are partially confirmed by the literature (e.g., negative genetic correlations between loading and crate scores: [11]) and our results, but partially not (e.g., positive phenotypic correlations between scale and load scores and vocal scores: [23]). Possibly, environmental influences on behaviour were particularly large in studies with low correlations, including our present study (e.g., insignificant correlations between FLS and FSS) conducted under practical farming conditions with e.g., different housing conditions. The assumption of a large impact of environmental factors is supported by the fact that correlations tend to be lower, and non-significant for older pigs, with which there was more time for environmental factors to act on the pigs. Also, possibly, the scoring scales used for recording behaviour did not reflect phenotypic variance sufficiently enough to yield reasonable correlations.

### Genetic parameters

The estimated heritabilities for behaviour traits in this study are comparable to the results of previous studies: The frequency of attempts to struggle recorded during the backtest was generally moderately to highly heritable [9, 30, 38]. In our study BT1 shows a lower heritability (h^**2**^ = 0.22 ± 0.10) compared to BT2 (h^**2**^ = 0.36 ± 0.08). D’Eath et al. [11] estimated heritabilities for load (24 h post mixing: h^**2**^ = 0.15 ± 0.02; end of test period: h^**2**^ = 0.16 ± 0.02) and scale scores (24 h post mixing: h^**2**^ = 0.17 ± 0.03; end of the test period: h^**2**^ = 0.10 ± 0.02). Another study [10] found for scale activity score h^**2**^ = 0.23, which is comparable to our results for fattening pigs’ scale scores (h^**2**^ = 0.20 ± 0.07), but considerably higher compared to our remaining results regarding scale-related behaviour (h^**2**^ = 0.08 ± 0.06 (RLS) and h^**2**^ = 0.02 ± 0.03 (FLS) for load scores as well as for scale scores h^**2**^ = 0.07 ± 0.07 (PSS) and h^**2**^ = 0.12 ± 0.05 (RSS)). Due to very low additive genetic variances INJ, PSS, RLS, and FLS seems to be not useful for breeding issues. These low additive genetic variances also need to be kept in mind when interpreting the genetic correlations of these traits with other traits. For example, while the correlation between INJ and PDG appears to reveal a remarkable genetic link at the first glance, this link might as well be non-existent as both traits lack an adequate additive-genetic basis. Similarly, great differences for heritabilities of weight gain during different stages of pigs’ life appear to be mainly due low additive genetic variances of this trait during preweaning and rearing, (σ^**2**^_A_ = 112.1 and σ^**2**^_A_ = 79.5, respectively) compared to fattening (σ^**2**^_A_ = 5707.2; see Table 4), although environmental factors such as differences in feeding might also have affected the results.

In contrast, to INJ, PSS, RLS, and FLS, the extent of skin lesions shows reasonable heritabilities. The heritabilities ranged from h^**2**^ = 0.22 ± 0.07 [24] to h^**2**^ = 0.43 ± 0.04 [39]. However, in the present study heritabilities for skin lesion scores were lower (LS1: h^**2**^ = 0.14 ± 0.06; LS2: h^**2**^ = 0.12 ± 0.07) and within the range of results found for direct observations of aggressive behaviour (attacks: h^**2**^ = 0.11 ± 0.07 to h^**2**^ = 0.29 ± 0.13 / reciprocal fights h^**2**^ = 0.04 ± 0.07 to h^**2**^ = 0.33 ± 0.07, [40]). Earlier results suggested that lesion scores recorded shortly after mixing mostly reflect the number of attacks received, but not of being an initiator of aggressive interactions [25]. This might explain slightly lower heritabilities for LS2 rather than LS1, since LS2 is more dependent on the behaviour of other animals in a group, which is confirmed by a similar trend in heritabilities of lesions evaluated 24 h and 3 weeks post mixing [39]. Interestingly, and considering the part-whole relationship, genetic correlations between lesion scores recorded before and after mixing were only at a medium level. Considering the same traits recorded repeatedly over time as separate traits is commonplace when applying random regression methodology [41, 42], and results indicate that, although the same trait is observed at a short interval, the genetic background differs considerably between these two points in time. This could be e.g., because different genes are switched on and off [43] in the different situations (fights during feeding which may mostly contribute to LS1 versus fights during mixing which are probably mostly responsible for the changes observed in LS2 compared to LS1) or between the different environments (in this case housing during rearing versus finishing). It could also be argued, that higher heritabilities and relationships between repeated recordings of skin lesions could have been obtained, if lesions had been scored separately for different body parts, but our previous results [25] from the same population of pigs does not confirm results from earlier studies [39] indicating that only lesions in the front are representative of fights. Taken together, the results show that selected behaviour or indicator traits might be useful for breeding purposes based on their heritabilities, but further aspects (e.g. feasibility of large-scale data collection, validity of the traits, partly antagonistic relationships with performance traits; see Table 6) have to be taken into account before using these traits for breeding.

Also based on the present data, limited conclusions can be drawn from genetic and phenotypic correlations among behaviour and performance traits. Owing to the small sample size, generally standard errors for genetic correlations were rather high, frequently resulting in correlations that are not significantly different from zero (Table 6). However, phenotypic and genetic correlations between skin lesions and daily gains indicate that higher levels of involvement in fights tend to be associated with better performance (LS1 & RDG: r_p_ = 0.15; r_g_ = 0.43 ± 0.25; LSD & FDG: r_p_ = 0.12; but r_g_ -0.27 ± 0.61). Although a pronounced relationship between skin lesions and individual aggressiveness is not unambiguously detected (e.g., [24, 25]), pigs with more skin lesions were at least involved in reciprocal fights either as the attacking or as the attacked pig, and likely the limited access to feed led to increased numbers and/or severity of fights. This gives evidence that these pigs with more skin lesions might have better weight gains due to higher assertiveness at the feeder.

### Linear versus binary assessment

In livestock research, and particularly, when on-farm research is conducted, behaviour traits are commonly evaluated via frequencies or scores. These scores are finite, discrete variables, which frequently exhibit substantial deviations from the statistical properties of Gaussian distributions. Nevertheless, the use of statistical and quantitative genetic methods assuming Gaussian properties is commonplace for such data (e.g., [33, 40]). Results of the present comparison of treating the scores either as Gaussian or as binary trait are in line with earlier research both at the phenotypic [44] as well as the genetic level [45], showing that parametric methods are robust against deviations from normality and that the latter (Gaussian) approach seems to be justified, and yields, for most variables comparable results as indicated by heritabilities that generally differ only by a small amount. Treating data as binary variables with roughly equal proportions of both behaviour categories would, based on calculations by [27] thus likely not greatly reduce genetic gain with moderately heritable traits, and indeed, in the present study, cross validation for the sample trait FSS suggested a slight superiority of the logit model.

In previous studies, these cross-validation approaches generally yielded satisfactory agreement between both approaches, although model performance may differ between animals of the two different trait categories, such that one model performs better for prediction of affected animals (or in our case animals exhibiting a stronger level of behaviour), while others perform better for prediction of unaffected animals [43, 46]. Also, results may be influenced considerably by the frequency distribution of the binary trait. In the present study, cut-off points for 0 and 1 codings were chosen to achieve roughly equal proportions of the two alternative responses, and it should be kept in mind, that these cut-off points may not be the best reflection of the biological background. Alternative cut-off points, on the other hand, with marked differences in proportions, will greatly influence levels of the transformed heritabilities [27].

Thus, recording for most traits could be simplified to binary responses, but cut-off points need to be carefully chosen, and it remains questionable, if advantages such as savings in labour by reducing the levels of behaviour categories outweigh costs e.g., of increased computational effort.

### Implications

Breeding of calm and less-aggressive pigs could contribute to improved animal welfare as well as lower stress levels for stockmen when handling pigs. Identification of suitable behaviour traits related to handleability and/or aggression is the first important step for integration of these characteristics into breeding programmes. The results of the present study show that there were no useful associations between the modified backtest or behaviour during iron injection as early indicators for behaviour or skin lesions (indicator for aggressions) assessed later in pigs’ life. Therefore, with regard to the investigated traits, direct recording of behaviour traits of interest at the specific point in pigs’ life rather than the use of indicator traits appears to be the most promising strategy when implementing behaviour traits into breeding programmes. Simplification of behaviour recording to binary traits appears to be possible, and may in some cases achieve superior results.
